# Rectal Prolapse Surgery: Balancing Effectiveness and Safety in Abdominal and Perineal Approaches

**DOI:** 10.7759/cureus.69868

**Published:** 2024-09-21

**Authors:** Imad Al Zangana, Rania H Al-Taie, Sajjad Al-Badri, Mustafa Ismail

**Affiliations:** 1 Department of Surgery, Medical Help Hospital, Erbil, IRQ; 2 Department of Surgery, University of Mustansiriyah, College of Medicine, Baghdad, IRQ; 3 Department of Surgery, University of Baghdad, College of Medicine, Baghdad, IRQ; 4 Department of Surgery, Baghdad Teaching Hospital, Medical City Complex, Baghdad, IRQ

**Keywords:** effectiveness, laparoscopic rectopexy, perineal approaches, rectal prolapse, surgical outcomes

## Abstract

The event in which the entire thickness of the rectum protrudes through the anal canal is called rectal prolapse. This ailment is common in the elderly population and especially in females. It causes some disastrous symptoms, including incontinence to feces and flatus, constipation, and discomfort, because of the weakness in the anorectal junction, making it mandatory for surgical correction. Over time, several surgical techniques have been developed; these are broadly classified into two categories: abdominal and perineal techniques. However, the best approach for surgery that minimizes recurrence while maximizing patient quality of life is still up for debate. A comprehensive review was conducted adhering to the Preferred Reporting Items for Systematic Reviews and Meta-Analyses (PRISMA) guidelines; a systematic search of the PubMed Database was performed to identify studies published between 2000 and 2024 with the keywords ((Rectal Prolapse) AND ("Perineal" OR "Laparotomy")). The inclusion criteria were focused on studies comparing the outcomes between surgical approaches at the abdominal and perineal locations, particularly on the recurrence rate, postoperative complications, and functional outcomes. In total, 21 studies were included in the review: these ranged from retrospective analysis and prospective studies to a multicentric randomized trial. In this review, abdominal approaches, particularly in the form of laparoscopic rectopexy, consistently demonstrated improved results compared to perineal techniques, with a much lower recurrence rate. The rates of mortality and morbidity were also remarkably lower in laparoscopic operations, which were advocated for suitable patients. However, perineal approaches, while still producing higher rates of recurrence, are a valuable alternative for elderly and high-risk patients due to their being relatively less invasive.

Laparoscopic rectopexy can be considered a better surgical method for rectal prolapse, as it has a lower recurrence rate and better functional outcomes. In contrast, perineal approaches will have their place in the management of rectal prolapse, given patient selection for patients at high risk with regard to surgery. Future research should be directed toward multicenter trials with long-term outcomes in order further to fine-tune surgery strategy and criteria for patient selection.

## Introduction and background

Rectal prolapse is defined as a full-thickness protrusion of the rectum through the anal canal. It peaks in the seventh decade of life, occurring more commonly in women. Indeed, some studies showed that nearly 50% of affected individuals were older than 70 years of age [1]. The issue of surgical management of rectal prolapse has remained complicated and controversial ever since, due to its multifactorial nature with deep ramifications on quality of life among patients.

Rectal prolapse is associated with severe symptoms, including fecal incontinence, constipation, and discomfort from prolapsing tissue, and must therefore offer sufficient indication for surgical intervention to return patients to regular function and quality of life. Numerous techniques have been developed and perfected over the last decades; they can be generally divided into two types of procedures: one that involves an abdominal approach and another involving a perianal approach. Each of them has a set of advantages and limitations.

In the abdominal region, open or laparoscopic techniques are generally preferred for the young and healthy patient; for all anatomical reasons, because of the recurrence acquired, and good functional results expected, as well. Open abdominal surgeries are most often related to a longer recovery period, more postoperative pain, and higher morbidity. On the other hand, laparoscopy has been more and more welcomed, mainly because its minimally invasive nature guarantees minimal pain after surgery, shortened postoperative lengths of hospital stay, and short recovery times. Among these, laparoscopic ventral mesh rectopexy (LVMR) stands out as a low-morbidity operation that preserves the function of the pelvic floor with minimal postoperative constipation and lower recurrence rates [2,3].

Perianal approaches, such as Delorme's and Altemeier's procedures, are usually reserved specifically for more elderly, debilitated patients with significant comorbidities. They are far less invasive, with reduced perioperative risks, but they generally have a higher recurrence rate compared to the abdominal approach. They provide a better alternative for the important management of high-risk patients with surgery than those who appear weak or unable to withstand general anesthesia [2,3]. The decision to use a perianal or an abdominal approach should be patient-oriented because this will more often be based on age, comorbidity profiles, and severity of the prolapse.

Thus, while abdominal procedures, might give better long-term results concerning functional improvement and recurrence, comparative analysis has also shown that perineal approaches may often be preferred in patients with a high surgical risk owing to the lower perioperative morbidity and the possibility of performing them under locoregional anesthesia. Further evolution of surgical techniques combined with the use of new biomaterials will no doubt improve the safety and efficacy of these procedures. [2]. It has been compared to show the best surgical strategies in relation to recurrence rate, postoperative complications, and quality of life after surgery, among different populations.

## Review

Methods

**Figure 1:**
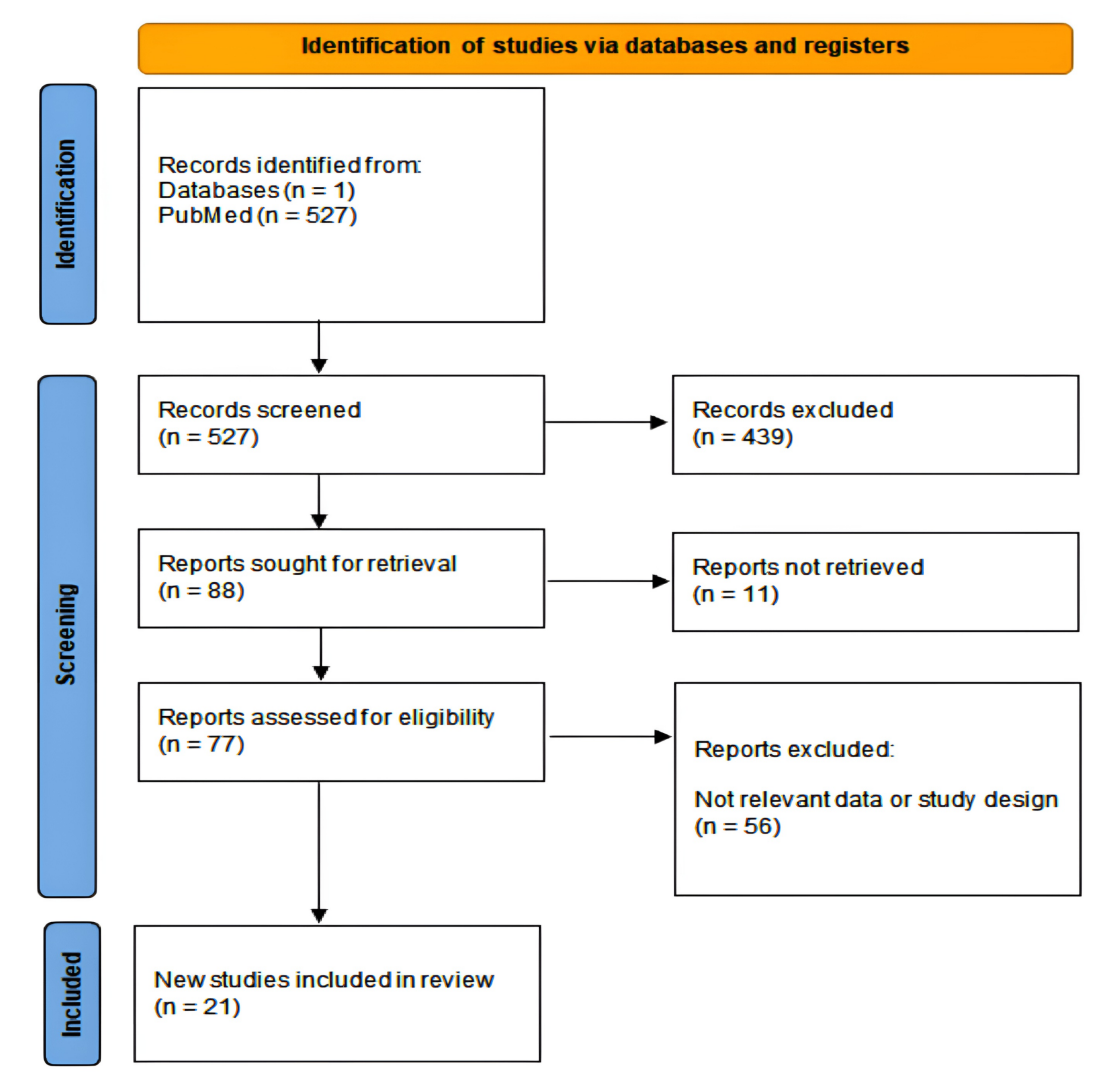
A PRISMA flowchart outlining the study selection process PRISMA: Preferred Reporting Items for Systematic Reviews and Meta-Analyses

This systematic review was conducted to critically appraise and compare the outcomes of abdominal (open and laparoscopic) and perineal surgical approaches in the management of rectal prolapse. Preferred Reporting Items for Systematic Reviews and Meta-Analyses (PRISMA) guidelines for systematic reviews and meta-analyses have been followed in this review (Figure 1) [4].

Search Strategy

A systematic search of the PubMed database was conducted for studies published from January 2000 to August 2024. The search strategy used was: ((Rectal Prolapse) AND ("Perineal" OR "Laparotomy")). These terms were combined in the search strategy with the purpose of looking for studies regarding the treatment of rectal prolapse with perineal approaches or abdominal, including laparotomy and laparoscopic procedures.

Inclusion and Exclusion Criteria

The specific focus of this review was comparative studies that researched the surgical management of rectal prolapse in adults. The interventions in these selected studies had to be comparisons of the perineal approach with abdominal surgery, including both open methods. Relevance and quality were considered in this review, focusing on a specific publication time frame from 2000 to 2024. Additionally, studies were required to report in the English language and represent relevant clinical outcome measures such as recurrence rates, postoperative complications, quality of life for patients, and functional outcomes. On the other hand, the review excluded studies not dealing with the surgical management of rectal prolapse; other types of studies, such as other aspects of the management of rectal prolapse, case reports, reviews, and studies with less than 10 patients, were excluded. Non-English publication titles were also removed from the list to maintain the review process.

Data Extraction

Data from the selected studies were independently extracted by two reviewers. The extracted data included study design, sample size, patient demographics, type of surgical procedure, recurrence rates, postoperative complications, patient quality of life, and functional outcomes. Any discrepancies between the reviewers were resolved through discussion until a consensus was reached.

Quality Assessment

The quality of the included studies was assessed using the Risk Of Bias In Non-randomised Studies - of Interventions (ROBINS-I) for cohort studies and the Cochrane Risk of Bias Tool for randomized controlled trials [5]. Based on their scores, studies were categorized as low, moderate, or high quality. The quality of the randomized controlled trials (RCTs) included in this review was evaluated using the Cochrane Risk of Bias 2 (RoB 2) tool [6]. The studies were categorized as low risk, some concerns, or high risk of bias based on the RoB 2 criteria.

Data Synthesis

A qualitative synthesis of the findings was performed, comparing the outcomes of abdominal (open and laparoscopic) and perineal approaches. The results were presented in both tabular and narrative forms, with a focus on key outcomes such as recurrence rates, postoperative complications, and functional results.

Results

This systematic review incorporated 10 studies assessing the outcomes achieved from different surgical procedures aimed at treating rectal prolapse, either by abdominal or perineal approach. The designs of the studies, which showed a retrospectively to prospectively conducted investigation, even comprised a multicenter randomized trial. Most of the patient populations were elderly, with a remarkable predominance of female patients, reflecting the higher incidence of rectal prolapse in older women.

After screening the titles and abstracts of these studies, full-text articles were retrieved for those that appeared to meet the inclusion criteria. Upon further evaluation, 21 studies were included in the review (Table 1) [7-27].

**Table 1:** Efficacy and safety of abdominal vs. perineal surgical techniques in rectal prolapse ASA: American Society of Anesthesiologists; CCIS: Cleveland Clinic Incontinence Score; DR: Delorme’s repair; KESS: Knowles‐Eccersley‐Scott‐Symptom; LOS: length of stay; LR: laparoscopic rectopexy; LRR: laparoscopic resection rectopexy; LVR: laparoscopic ventral rectopexy; LVMR: laparoscopic ventral mesh rectopexy; OR: open rectopexy; ORR: open resection rectopexy; PRS: perineal rectosigmoidectomy; PSPR: perineal stapled prolapse resection; PSR: perineal stapler resection; QoL: quality of life; RRP: resection rectopexy; SF-36: 36-Item Short Form Survey; UTI: urinary tract infection

Study ID	Author	Country	Year	Study design	Sample size	Patient demographics	Surgical approach	Type of procedure	Primary outcomes	Secondary outcomes	Follow-up duration	Complications	Key findings
1	Sobrado et al. [7]	Brazil	2004	Retrospective study	51	Mean age: 56.7 years, 39% females	Abdominal, perineal	Presacral rectopexy, sigmoidectomy, rectosigmoidectomy, levatorplasty	Recurrence rates	Fecal incontinence, constipation	49 months	Sacral bleeding, rectovaginal fistula	Low recurrence rates with both approaches, with the abdominal approach having higher morbidity but fewer recurrences.
2	Kariv et al. [8]	USA	2006	Case-control study	111 (LR), 161 (OR)	Mean age: 56.8 years, 87% female	LR and OR	Posterior mesh fixation, sutured rectopexy	Recurrence rates	Functional outcomes (constipation, incontinence)	56 ± 40 months for LR vs 63 ± 41 months for OR	Conversion to open surgery (7.2% in LR), minor complications similar between groups	The LR group had shorter hospital stays and reduced constipation postoperatively compared to the OR group. No significant difference in recurrence rates between LR and OR.
3	Hoel et al. [9]	Norway	2008	Retrospective study	56	Median age: 59 (abdominal) 78 (perineal)	Abdominal, perineal	Rectopexy, resection rectopexy, Delorme-Thiersch repair	Recurrence, patency	Constipation, anal insufficiency	Up to 25 years	20% overall complications	Abdominal rectopexy had better patency, but perineal approach had higher recurrence.
4	Wijffels et al. [10]	UK	2010	Retrospective study	80	Elderly patients (age ≥ 80), mixed ASA grades	Laparoscopic and perineal	LVR	Mortality, recurrence rates	Length of stay, functional outcomes (continence)	Median 23 months	No mortality, 13% had minor complications (e.g., chest infection, UTI)	LVR is a safe and effective procedure for treating full-thickness rectal prolapse in elderly patients, showing a significantly lower recurrence rate compared to perineal approaches.
5	Riansuwan et al. [11]	USA	2010	Retrospective study	177	Mean age: 69 (perineal), 55 (abdominal)	Abdominal, perineal	Abdominal: Sutured, mesh, resection rectopexy; Perineal: DR, Altemeier procedure	Recurrence rates	QoL (SF-36), constipation (KESS), incontinence (CCIS)	3.9 years (AO), 3.1 years (PO)	No significant difference in complications	Abdominal approach had better physical QoL outcomes and lower recurrence rates, Perineal approach was suitable for elderly patients with comorbidities.
6	Lee et al. [12]	USA	2011	Retrospective study	131 (LAP: 8, PRS: 123)	Elderly patients (LR: mean age 71 years, PRS: mean age 80.7 years)	Laparoscopic and perineal	LR vs. PRS	Recurrence rates, operative time, blood loss	Length of hospital stay, postoperative complications	Mean follow-up 12.8 months for PRS, 6.9 months for LR	Higher blood loss and longer operative time in LR group; similar hospitalization length	LR is safe and feasible in elderly patients but has a longer operative time and higher blood loss compared to PRS. Recurrence rates were comparable between both groups.
7	Fang et al. [13]	USA	2012	Retrospective Study	1,469	Adults, ASA classifications 1-4, age >80	Abdominal and perineal	Open abdominal, laparoscopic, perineal	Mortality rates	Procedure selection based on age and ASA class	30 days	Higher mortality in perineal procedures, particularly in high-risk groups	Perineal approaches are associated with higher mortality in high-risk patients compared to abdominal approaches. Laparoscopy shows advantages in lower mortality rates.
8	Mustain et al. [14]	USA	2013	Propensity-matched cohort	1,126	Mixed, elderly, higher risk in perineal group	Abdominal and perineal	Abdominal (laparoscopic,open); Perineal (Altemeier's procedure, DR)	Mortality, major morbidity	Operative time, length of stay, specific complication rates	30 days	Similar major morbidity rates (8.9% abdominal, 6.4% perineal); no significant difference in postoperative complications	The study found no significant difference in major morbidity between abdominal and perineal approaches. Mortality rates were equivalent. Many patients who undergo perineal repair could safely be treated with abdominal surgery without an increase in complications.
9	Senapati et al. [15]	UK, India, Spain, Serbia, Finland	2013	Randomized controlled trial	293	Mean age 63 years	Abdominal and perineal	Abdominal: Suture vs RRP, Perineal: Altemeier’s procedure, DR	Recurrence, bowel function, quality of life	Operative morbidity, mortality	3 years	Five treatment-related deaths	Abdominal and perineal procedures had similar recurrence and quality of life outcomes.
10	Mik et al. [16]	Poland	2014	Retrospective study	86	Mean age: 67, Female	Abdominal and perineal	Sutured rectopexy, Altemeier's procedure, DR	Recurrence rates	Functional outcomes (continence, sphincter function)	32 months	DR: 1.4% mortality, 11.8% recurrence	Perineal approaches had higher recurrence; Altemeier's procedure improved sphincter function significantly.
11	Makinen et al. [17]	India	2014	Prospective observational	27	Males: 46 years, females: 60 years	Abdominal and perineal	Posterior mesh rectopexy, DR	Recurrence rates	Incontinence improvement, constipation	14 months	Rectopexy: 17%, DR: 10%	Rectopexy had no recurrences but increased constipation.
12	Lee et al. [18]	South Korea	2014	Retrospective study	104	Adults, mean age AP: 52, PP: 67	Abdominal and perineal	Abdominal: rectopexy (open/laparoscopic), Perineal: DR, Altemeier's procedure	Recurrence rate, operative outcomes	Functional outcomes (constipation, incontinence)	Median 26 months (AP), 22 months (PP)	Higher recurrence in the PO group, similar complications between groups	AO group had longer operation time and hospital stay but lower recurrence. PO group had higher rates of persistent incontinence.
13	Young et al. [19]	USA	2015	Retrospective study	3,254	Older patients, mean age of 76 years in the PR group and had a higher mean ASA class (2.7) compared to patients undergoing laparoscopic (58 years, 2.2) and open abdominal procedures (58 years, 2.3).	Laparoscopic, open, perineal	LR, LRR, OR, ORR, PR	30-day morbidity, mortality	Operative time, length of stay, intraoperative blood transfusion	30 days	Lower morbidity in the PR group and higher mortality in PR group compared to LR	Laparoscopic approaches showed comparable morbidity to perineal surgery with lower 30-day mortality. Open abdominal procedures had higher morbidity compared to perineal surgery.
14	Elagili et al. [20]	USA	2015	Retrospective study	75	93% female, Mean age: 72	Perineal	DR, Altemeier's procedure	Recurrence rates	Postoperative stool frequency, QoL	13 months	Altemeier's procedure: 18% anastomotic leak	DR had higher recurrence, while Altemeier's procedure had more complications. Both improved QoL.
15	Ng et al. [21]	Australia	2019	Retrospective study	157	Predominantly elderly females (94%)	Abdominal and perineal	Rectopexy, resection rectopexy, DR, PRS	Recurrence rates (at 5 years)	Mortality, morbidity, length of hospital stay	Median 4.5 years (up to 16.5 years)	Higher recurrence in DR and PRS, comparable morbidity across groups	Abdominal approaches (rectopexy, resection rectopexy) had significantly lower recurrence rates at 5 years (5% and 3%) compared to perineal approaches (52% for DR, 30% for PRS). However, abdominal approaches had a tendency for higher morbidity.
16	Nacion et al. [22]	South Korea, Philippines	2019	Retrospective study	46	Mean age: 65.2 years	Abdominal and perineal	LR, DR, Altemeier's procedure	Recurrence rates	Hospital stay, morbidity	10 years	Abdominal: minor UTI, neurologic bladder	Abdominal approach showed no significant recurrence rate difference from perineal techniques.
17	Mohapatra et al. [23]	India	2021	Randomized controlled trial	58	Mean age: 39.8 years (open) 40.7 years (laparoscopic)	Abdominal	LR, OR	Postoperative pain, recurrence	Hospital stay, bowel resumption	18 months	One recurrence in the open group	LR had less pain and shorter hospital stays, with no significant recurrence difference.
18	Smedberg et al. [24]	Sweden	2022	Multicentre randomized trial	122	Predominantly elderly (mean age 71.3 years), 94% female	Abdominal and perineal	DR, Altemeier’s procedure, suture rectopexy, RRP	Recurrence rates, Wexner incontinence score, RAND-36 Quality of Life	Mortality, morbidity, operative time, length of hospital stay	Mean follow-up 2.6 years, long-term follow-up up to 10.1 years	Minor complications in both groups, but no significant difference in postoperative complications	Recurrence rates were higher for perineal approaches compared to abdominal approaches. Abdominal procedures had longer operative times but were associated with better long-term outcomes in terms of recurrence.
19	Hu et al. [25]	China	2022	Retrospective cohort study	51	Males only, Mean age: 27.3 years	Abdominal and perineal	LVR, DR, Altemeier’s procedure	Recurrence rates	Postoperative defecation, LOS, QoL	48.5 months	Perineal: 20.7%, abdominal: 0%	Abdominal repair had lower recurrence and complications, while perineal repair improved constipation.
20	Roy et al. [26]	Canada	2023	Retrospective review	44	Mean age 81 (PSPR), 74 (Altemeier)	Perineal	PSPR, Altemeier’s procedure	Operative time, recurrence rates	Length of stay, complications	6 months	PSPR: 20%, Altemeier’s procedure: 15.8%	PSPR was faster and more cost-effective, with similar recurrence rates.
21	Habeeb et al. [27]	Egypt, Italy, Spain	2024	Retrospective cohort study	330	Elderly, mean age: 68 years	Abdominal and perineal	LVMR, PSR	Recurrence rates	Functional outcomes (Wexner incontinence, Altomare constipation)	4 years	LVMR: 1.2% mortality, PSR: 5%	LVMR showed better recurrence outcomes and higher satisfaction compared to PSR.

Notably, no duplication of studies was found during the screening process. The quality of the studies was assessed using the RoB 2 tool for randomized trials (Table 2) and ROBINS-I for non-randomized studies (Table 3), showing an overall low to moderate risk of bias. Minor concerns were noted, but they did not significantly affect the reliability of the findings.

**Table 2:** Quality assessment of the included studies using the RoB 2 tool for randomized controlled trials RoB 2: Cochrane Risk of Bias 2

Study	Bias arising from the randomization process	Bias due to deviations from intended interventions	Bias due to missing outcome data	Bias in measurement of the outcome	Bias in selection of the reported result	Overall
Senapati et al. (2013) [15]	Low	Low	Some concerns	Low	Low	Low
Mohapatra et al. (2021) [23]	Low	Low	Some concerns	Low	Low	Some concerns
Smedberg et al. (2022) [24]	Low	Low	Some concerns	Low	Low	Some concerns

**Table 3:** Risk of Bias assessment of the included studies using ROBINS-I tool ROBINS-I: Risk Of Bias In Non-randomised Studies - of Interventions

Authors	Confounding	Selection of patients	Classification of interventions	Deviations from intended interventions	Missing data	Measurement of outcomes	Selection of reported results
Sobrado et al. (2004) [7]	Moderate	Low	Low	Low	Moderate	Serious	Moderate
Kariv et al. [8]	Moderate	Serious	Low	Low	Serious	Serious	Moderate
Hoel et al. (2008) [9]	Moderate	Low	Low	Low	Moderate	Moderate	Moderate
Wijffels et al. [10]	Moderate	Serious	Low	Low	Serious	Serious	Moderate
Riansuwan et al. (2010) [11]	Moderate	Low	Low	Low	Moderate	Serious	Moderate
Lee et al. [12]	Moderate	Moderate	Low	Low	Low	Moderate	Low
Fang et al. [13]	Moderate	Low	Low	Low	Serious	Serious	Moderate
Mustain et al. [14]	Moderate	Moderate	Low	Low	Serious	Serious	Moderate
Mik et al. (2014) [16]	Moderate	Low	Low	Low	Moderate	Serious	Moderate
Makineni et al. (2014) [17]	Moderate	Low	Low	Low	Moderate	Serious	Moderate
Lee et al. [18]	Moderate	Moderate	Low	Moderate	Low	Moderate	Serious
Young et al. [19]	Moderate	Low	Moderate	Low	Low	Moderate	Low
Elagili et al. (2015) [20]	Moderate	Low	Low	Low	Moderate	Serious	Moderate
Ng et al. [21]	Moderate	Moderate	Low	Low	Low	Moderate	Moderate
Nacion et al. (2019) [22]	Moderate	Low	Low	Low	Moderate	Serious	Moderate
Hu et al. (2022) [25]	Moderate	Low	Low	Low	Moderate	Serious	Moderate
Roy et al. (2023) [26]	Moderate	Low	Low	Low	Moderate	Serious	Moderate
Habeeb et al. (2024) [27]	Moderate	Low	Low	Low	Moderate	Moderate	Moderate

Recurrence Rates

On the whole, from the accruing evidence in the review studies, recurrence rates were an important outcome, and comparatively better results were shown for the various surgical modalities, yielding the best results for abdominal ones, especially in laparoscopic rectopexy. For example, the recurrence rate at five years was found to be significantly lower for rectopexy (5%) and resection rectopexy (3%), from a multi-center study conducted by Ng et al. in 2019 [21], in contrast to 52% and 30% for Delorme's repair and perineal rectosigmoidectomy, respectively. It, therefore, means that most of the pelvic conditions will be eliminated through the abdomen, especially if the laparoscopic measures are applied. Similarly, Smedberg et al. (2022) [24] have added further weight to the emerging evidential base that, despite being technically greater in complexity and longer in operative time, the open abdominal approach indeed was associated with considerably fewer cases of recurrence when placed under long-term surveillance. This had been supported by Lee et al. (2014) [18] because they had reported that although most of the perineal approaches had increased the recurrence and persistent incontinence, like that of Delorme's and Altemeier's repairs, the complication rates recorded are the same for both the abdominal and perineal approaches.

Bang Hu et al. (2022) [25] found that in patients who underwent laparoscopic ventral rectopexy (LVR), there was no recurrence, in contrast to 20.7% following perineal repairs involving Delorme's or Altemeier's procedures. In the report of Riansuwan et al. in 2010 [11], the recurrence rate was also significantly higher in the perineal group at 26.5%, compared with 5.2% in the abdominal group after three years of follow-up. Similarly, in 2014, Makinen et al. demonstrated that there was no recurrence in the group with abdominal rectopexy, while in the perineal group, it was 10% [17]. Similarly, Sobrado et al. (2004) [7] and Senapati et al. (2013) [15] have shown that although less injurious to elderly patients, the recurrence rates after perineal approaches are over threefold their abdominal route counterparts. Thus, taken in a nutshell, the evidence seems to indicate that abdominal approaches would ideally reduce recurrence rates, making the approach better suited for younger, fitter patients, whereas perineal approaches may be better suited and reserved for elderly patients who are considered poor candidates for abdominal surgery.

Mortality and Morbidity

Mortality and morbidity outcomes can provide important insights into the safety profile of the different surgical options. Fang et al. (2012) [13] also reported a significantly lower incidence of mortality rates with perineal procedures compared to abdominal in high-risk groups. Remarkably, it can be concluded that more fitness-for-purpose cases might be carried out for patients, as the stoma or abdominoperineal approach gives a significantly higher pattern of mortification curves compared to the minimally invasive laparoscopically related approach.

These findings were reiterated by Young et al. (2015) [19], and though the morbidity was equivalent to perineal surgery in laparoscopic procedures, they still evidenced lower 30-day mortalities. More serious morbidity was expressed in open abdominal procedures, solidly endorsing that laparoscopic approaches are more preferred when, of course, suitable. Mustain and colleagues in 2013 [14] utilized this design for a propensity-matched cohort design and found that major morbidity seemed to be no different abdominally versus using a perineal approach. With this study, even patients considered at high risk for perineal repair were considered to have the potential to pursue abdominal surgery without being at increased risk for complications.

The assessment of postoperative quality of life was consistent across multiple studies, with most reporting no significant difference in mental health outcomes between abdominal and perineal procedures. However, the physical component of quality of life was better in patients undergoing abdominal surgery, as shown in studies like Riansuwan et al. (2010) [11], where abdominal operation patients scored higher on the SF-36 physical functioning scale (39.6 vs. 33.0 for perineal procedures).

Senapati et al. [15] reported that both abdominal and perineal groups showed similar improvements in quality of life, though abdominal procedures had lower recurrence rates. Further, interestingly, patients undergoing perineal procedures, in particular in the high-risk group, showed acceptable long-term functional outcomes, underscoring that perineal approaches remain a valid option in elderly and comorbid patients.

Functional Outcomes

Functional outcomes, in particular improvements in constipation and incontinence, were pivotal in determining surgical efficacy. Kariv et al. [8] reported that laparoscopic rectopexy reduces constipation postoperatively, and its recurrence rates are similar to open rectopexy, a dual-benefit road to improvement in function and durability. However, Lee et al. [12] showed that while laparoscopic rectopexy was safe and could be performed on elderly patients, it carries the disadvantage of a longer operation time and higher blood loss compared to perineal rectosigmoidectomy. Notwithstanding, there was no large difference in recurrence between the two approaches; the above data go to show that functional outcomes post-laparoscopic rectopexy are satisfactory and are not compromised by patient safety.

Makinen et al. (2014) [17] found that rectopexy improved continence and reduced constipation in most patients, though it somewhat aggravated postoperative constipation in a few, while perineal procedures such as Delorme's and Altemeier's had a higher recurrence of incontinence despite improvement in continence for most patients.

Smedberg et al. (2022) [24] found no significant differences in quality of life or bowel function between abdominal and perineal approaches; however, their study emphasized the importance of long-term follow-up to monitor late recurrences and functional decline. The functional improvements noted with abdominal procedures, particularly regarding incontinence, make them the preferred approach in patients where long-term bowel function is a priority.

Complications

Complication rates were different among the different surgical approaches; this may also further help the choice of procedure according to individual patient and clinical factors. Presenting a range of laparoscopic approaches with careful patient selection and skilled surgical technique, this study suggests that excellent outcomes can be achieved with minimal complications. The study by Wijffels et al. in 2010 [10] added support to the evidence by showing that LVR is potentially safe and effective, with the site of recurrence after repair being significantly low and less complicated than with a perineal approach, especially in the elderly.

Sobrado et al. (2004) [7] reported sacral bleeding and rectovaginal fistula as complications in abdominal procedures, while Altemeier’s procedure had a high rate of anastomotic leaks (18%) in the study by Elagili et al. (2015) [20]. Despite a higher incidence of complications, abdominal approaches were often associated with improved overall patient outcomes. Roy et al. (2023) [26] found that perineal stapled prolapse resection (PSPR) had a complication rate of 20%, similar to Altemeier’s 15.8%, but PSPR was quicker and more cost-effective.

Perineal approaches were associated with fewer immediate perioperative complications, including blood loss and overall operative time, but these benefits were offset by higher recurrence rates and complications in the long term, such as persistent constipation and incontinence. These findings highlight individualized surgical planning as the key to optimizing benefits derived through minimally invasive techniques and at the same time reducing potential risks.

Summary of Key Findings

In conclusion, on the basis of findings from this systematic review, abdominal procedures appear to be superior to perineal repairs, with laparoscopic techniques being the best option for achieving lower recurrence and improving functional outcomes in the surgical treatment of rectal prolapse. However, while the perineal approaches have shown an increased recurrence rate and high-risk profile compared to abdominal surgery, they might be valuable options for those not tolerating abdominal procedures. The surgical technique to adopt should lie truly subject to a comprehensive assessment of patient specificity that includes age, associated comorbidities, and risk with regard to recurrence for maximal outcomes and aspects of patient security.

Discussion

The surgical aspect of rectal prolapse is unique because of the peculiar difficulties in deciding the best surgical approach to be used, which both accomplishes the goal and, at the same time, ensures patient safety. This review highlights differences in key outcomes between the abdominal open, laparoscopic, and perineal approaches that are pertinent to clinical decision-making. This review also integrates contemporary studies and historical perspectives in bringing focus to how treatment strategies have evolved over time.

Recurrence emerged as an important measure of outcome when comparing the two approaches in this review: abdominal approaches, specifically laparoscopic rectopexy, led to uniformly improved outcomes over perineal techniques. Individual studies, such as those published by Ng et al. in 2019 [21] and Smedberg et al. in 2022 [24], further informed that abdominal procedures, especially laparoscopic, have a significantly lower recurrence rate, underpinning their role as the preferable surgical option for appropriate candidates.

Across almost all studies, recurrence rates were substantially lower in the abdominal surgery group compared to the perineal group. For instance, Hu et al. (2022) [25] and Riansuwan et al. (2010) [11] both showed marked differences, with recurrence rates for abdominal procedures as low as 0%-5%, compared to up to 26.5% for perineal surgeries. This aligns with established literature, which also supports the idea that the anatomical fixation offered by abdominal procedures, particularly mesh-based rectopexy, provides a more robust long-term solution for rectal prolapse. Moreover, according to representatives of evidence-based medicine studies, such as Makinen et al. (2014) [17] and Mohapatra et al. (2021) [23], the benefits of laparoscopic approaches include minimizing recurrence and all the benefits of minimally invasive surgery, such as less postoperative pain and quicker recovery.

This preference is further supported by the international survey conducted by Jonkers et al. (2012) [28], which revealed that LVR is widely favored in Europe, while laparoscopic resection rectopexy is more commonly employed in North America. However, the historical context provided by Hunt et al. (1985) [29] offers a contrasting perspective. In this study, silastic rings were utilized to treat rectal prolapse in elderly patients deemed to bear high risks for abdominal surgery. While this procedure yielded results inferior to what is normally seen with abdominal procedures, it did provide a manageable alternative for patients unfit for laparotomy. This early innovation underscores how problems associated with managing rectal prolapse in high-risk populations have persisted and highlights the need for individual treatment strategies.

The review also presents how there is a variance in mortality and morbidity through differences in surgical methods. Among those that underwent laparoscopic procedures, the mortality rates proved continuously low compared to the open abdominal and perineal procedures, as outlined by the studies conducted by Fang et al. (2012) [13] and Young et al. (2015) [19]. These results become highly relevant when applied in high-risk patients, in whom opting for a less invasive, safer surgical strategy will significantly tilt the balance in favor of an improved overall outcome. The international questionnaire complements this well, as the outcomes of surgical choices are found to be ever so markedly influenced by patient demographics, reflecting the results of this study clearly [28]. The bulk of the responders favored perineal methods in the case of the frail or very old, perhaps reflecting the high proportion of respondents who favored more cautious surgical behavior, balancing invasiveness with patient safety. Functional results, in particular the improvement of constipation and incontinence issues, are very important during surgery for rectal prolapse because they bear direct implications for the quality of life of the patient after surgery.

These indicate that significantly better enhancement of the result occurs for laparoscopic rectopexy than for postoperative constipation-based open rectopexy, in which Kariv et al. (2006) [8] had favorable outcomes in this respect. Still, long temperature times associated with the procedure and high intraoperative loss of blood count against enhanced improvement and argue that much caution needs to be taken in the selection and perioperative management of patients.

While less overall effective, the use of silastic rings substantially improved continence for those whose prolapse was adequately controlled. This residually important function outcome in an average 80-year-old patient population resonates with understanding the importance of assessing outcomes in prolapse surgery on a continent whim.

Whereas abdominal approaches are generally more effective in preventing recurrence, they also have higher intraoperative demands, with longer operative times and greater blood loss noted, as in studies by Riansuwan et al. (2010) [11] and Sobrado et al. (2004) [7]. Complications such as rectovaginal fistula and sacral bleeding occur more commonly with these approaches, although they have generally been rare and well-managed with contemporary surgical techniques. The perineal approaches tend to have fewer associated perioperative risks.

Studies such as those by Elagili et al. (2015) [20] and Roy et al. (2023) [26] highlight the reduced blood loss, shorter hospital stays, and quicker recovery times associated with perineal techniques. However, the downside of these approaches is a higher incidence of postoperative complications related to functional outcomes, particularly recurrence of prolapse and issues with constipation and incontinence.

The abdominal approach, especially in the case of offered rectopexy, was a better outcome for continence and constipation, according to studies performed by Mik et al. (2014) [16] and Makinen et al. (2014) [17]. The obvious advantage of rectopexy in improving long-term bowel function seems to lie in its capacity to address both the structural and functional components of prolapse. On the other hand, while perineal procedures are effective in addressing prolapse, they are usually less reliable in the resolution of associated functional issues.

As noted in studies like those by Elagili et al. (2015) [20] and Smedberg et al. (2022) [24], perineal procedures tend to have higher rates of persistent or recurrent incontinence and constipation. Delorme’s procedure, for example, is associated with a relatively high risk of recurrence of incontinence, though it offers relief from the bulk symptoms of prolapse in the short term.

The data from the survey add weight to this by pointing out that 56% of those polled work in multidisciplinary teams for the management of rectal prolapse [28]. Such a team may also include a multidisciplinary care team that consists of other experts in addition to the surgeon, including but not limited to gynecologists, radiologists, and pelvic floor physical therapists, among others, who pool their resources together, potentially meaning the additionality of value addition to care in rectal prolapse characterized by attending to the multifactorial. An article by Vogel et al., 2020 [30] presents one of the most robust studies globally to explore gender disparities in rectal prolapse surgery, with a finding that males with rectal prolapse are worldwide younger and have a low surgical risk profile compared with their female counterparts.

This difference is important when it comes to surgical approach, with younger, healthier males more apt for laparoscopic or open procedures, while older females more commonly receive perineal procedures. No significant differences in postoperative morbidity, mortality, or reoperation rates based on sex were observed for the surgical approach. They did, however, take note of the fact that the males who received a laparoscopic procedure did have higher rates of venous thromboembolism and respiratory complications. It is important to consider the individual risk factors and not just gender on its own in relation to outcomes during postoperative care. The dispensation of the therapeutic approach implemented for the treatment of rectal prolapse, especially the use of robotic ventral rectopexy, appeared very promising.

As presented by Formisano et al., relevance vector regression (RVR) has specific benefits derived from the great mathematical dexterity, enhanced visualization, and better dissection made with more precision in the deep and tight pelvic operational field spaces than those with LVR [31]. Operative times are indeed significantly longer, although overall recurrence rates are comparable, and the functional results are better-fewer cases with postoperative constipation and fecal incontinence. Although the robotic costs run much higher, the decreased rates of complications and shorter length of stay in the hospital probably render the overall cost more reasonable. There is a more profound implication with this: in the surgical management of rectal prolapse, RVR must still be publicized as absolutely being on the table, most especially in those cases that are somewhat more complex, when the conventional approaches tend to be less cost-effective. Recent work has tried to use traditional Chinese herbal medicine, especially Buzhong Yiqi decoction, in the treatment of rectal prolapse through surgery. Xie et al. (2020) [32] prepared a systematic review and meta-analysis protocol to assess the therapeutic effect and safety of the two in combination. The outcome reveals the effectiveness of such an approach in surgery when combined with Buzhong Yiqi decoction and can be realized through an increase in muscle tone, a lowered rate of recurrence, and the relief of such symptoms as fecal incontinence. This reveals the possible benefits that might derive from the association of traditional medicine with common surgical methods especially afforded for the management of chronic and recurrent cases of rectal prolapse. Yet evidence supporting these findings with the need to increase high-quality randomized controlled trials to address the observed heterogeneity in the literature is clear in this study. Surgical management of rectal prolapse has changed hugely, with the recognition of minimally invasive approaches. Ng et al. (2021) [33] offer a good broad overview of these trends, noting that the use of ventral mesh rectopexy (VMR) has been on the rise because of a nerve-sparing approach and better results. The study found an increasing trend towards the preference of a VMR operation in younger, fitter, and less comorbid patient groups, whereas the older or more frailer patient groups would more likely get a perineal procedure, for example, Delorme's. Nevertheless, insofar as symptom relief with the recurrence rate of rectopexy is concerned, both were similar between the two methods of the surgical approach. Accordingly, this study underscores the important necessity of the customization of surgical approaches toward individual patient profiles and the trend for the increasing acceptance of minimally invasive techniques in the Asian context. Above all, that of the surgical repair of rectal prolapse in children, particularly burdensome and characterized by already different indications from those in the adult patient.

Saadai et al. (2020) [34] found, that despite the majority undergoing spontaneous resolution or recovering with conservative treatment, a selected few recovered with symptomatic lesions that demanded surgery. The variation in surgical techniques includes sclerotherapy, Thiersch's cerclage, or rectopexy, depicting an unstandardized model in pediatric care. Decision to opt for a surgical treatment is mainly influenced by the experience of an operator and underlying conditions in a patient, where rectopexy usually has good outcomes with approximately 5% to 10% of recurrence rate. This signifies that the treatment approach for pediatric rectal prolapse needs to be individualized, and a possible need to escalate into more tailored protocols exists. The management of recurrent rectal prolapse also poses another problematic surgical issue for which there is no uniform stand on which procedure to take. Chung et al. (2023) [35] compared the outcomes of abdominal and perineal methods of approach in surgery among patients presenting with recurrence of the rectal prolapse problem. They found that both are useful but each has its advantages; in the abdominal method, particularly in the laparoscopic ventral rectopexy, which involved long-standing operation times and hospital stays, there were no significantly different recurrence rates and postoperative complications compared with the perineal approach. Such results confirm the idea that surgical decision-making should be tailored to the state of the patient according to age, comorbidities, and surgical history. The overall data from the primary evidence reviewed indicates that the clinical implications are several.

First, among suitable candidates for the procedure, laparoscopic rectopexy should be the first-line surgical option for rectal prolapse, with low recurrence, good functional results, and low morbidity. However, the role of perineal approaches, including those described in the older studies, still has an important role for elderly people or individuals at high risk, in whom a safer performed operation is likely less invasive than with true recurrence aimed at. Moreover, the strategy in surgical management has to be individualized, factoring in variables such as patient's age, comorbidities, and overall surgical risk. The results of rectal prolapse surgery can therefore be optimized to maximize the well-being of patients if minimally invasive facilities are combined with appropriately instituted perioperative management. The survey suggests that the present variability in practice should be ironed out by set guidelines and also by prospective comparative studies, in order to ameliorate common treatment outcomes in different parts of the country. The limitations of this review are to be cited, however.

The variability in the designs of the studies, with different study populations, patient characteristics, and different follow-up durations, can introduce heterogeneity that affects the generalization of the findings. Additionally, the retrospective nature of many of these studies could be associated with selection bias and incomplete data reporting, potentially influencing the observed outcomes. Even the historical data provided, however valuable it proves in the context, does not really mean much for present-day practices with respect to surgery. In conclusion, this review accents the superiority of laparoscopic rectopexy in the management and treatment of rectal prolapse, mainly with regard to recurrence rates and functional outcomes, but at the same time, it continues to acknowledge the ongoing role of perineal approaches in specified patient populations.

Therefore, the integration of historical perspectives, such as treatment with silastic rings, into the study of modern therapeutic approaches mirrors changes over the years in practice and indeed the important fact that care of the patient requires an individualistic approach. Therefore, prospective, long-term, multicentre randomized trials are requested in order to receive further clarification of the comparative effectiveness of these surgical techniques and further, evidence of patient selection criteria refinement in order to optimize the outcome.

## Conclusions

Laparoscopic rectopexy has gained winning arguments as the surgical modality in rectal prolapse, with better recurrence rates and superior functional results compared with the perineal techniques. The evidence taken as a whole favors minimal abdominal invasive approaches, though perineal procedures remain important in the management of the older and high-risk patients. Additional research should focus on prospective randomized trials comparing surgical techniques in a long-term setting, including multiple centers, recurrence, and optimal functional outcome with patient quality of life as the main interest. In addition, smooth standardization of guidelines and research on the use of new technologies in surgery, such as robots, is mandatory for further evolution in treatment strategies and the enhanced quality of patient care in rectal prolapse management.
